# Evaluation of the Accuracy of Horse Body Weight Estimation Methods

**DOI:** 10.3390/ani10101750

**Published:** 2020-09-26

**Authors:** Wanda Górniak, Martyna Wieliczko, Maria Soroko, Mariusz Korczyński

**Affiliations:** 1 Department of Environment Hygiene and Animal Welfare, Wroclaw University of Environmental and Life Sciences, Chelmonskiego 38C, 51-630 Wroclaw, Poland; mbarabasc@gmail.com (M.W.); mariusz.korczynski@upwr.edu.pl (M.K.); 2 Institute of Animal Breeding, Wroclaw University of Environmental and Life Sciences, Chelmonskiego 38C, 51-630 Wroclaw, Poland; maria.soroko@upwr.edu.pl

**Keywords:** horses, breed, body weight estimation, estimation formula

## Abstract

**Simple Summary:**

Horse body weight estimation and monitoring of the weight variations are necessary to determine the amount of feed and feed additives for the proper functioning of the animal. Due to the cost and practical challenge of weighing horses on a large scale, several alternative methods for estimating the body weight of horses have been developed. One of them is to determine a horse’s body weight using a formula. The aim of the study was to evaluate established formulae for estimating horse body weight from data gathered using measurement tape. The investigation was conducted on a group of 299 adult horses of five breeds: ponies, Polish Noble Half Breed, Silesian Breed, Wielkopolski Breed and Thoroughbred. For each horse, body measurements were performed and the actual body weight of the horses was measured with an electronic scales. The horse’s body weight measurements were compared with the result of seven different formulae. It was found that the use of formulae for body weight estimation can be useful in determining feed dosages and additives, medicines, or deworming agents.

**Abstract:**

Methods of estimating horse body weight using mathematical formulae have better accuracy than methods of reading body weight from measuring tape. The aim of the study was to evaluate established formulae for estimating horse body weight from data gathered using measurement tape. The research was conducted in a group of 299 adult horses and ponies of selected breeds: ponies (*n* = 58), Polish Noble Half Breed (*n* = 150), Silesian Breed (*n* = 23), Wielkopolski Breed (*n* = 52), and Thoroughbred (*n* = 16). Body measurements were performed on each horse using a measuring stick and tape. The actual body weight of the horses was measured with electronic scale. Statistical analysis was carried out separately for individual breeds of horses. In each of the research groups formulae were selected, the results of which were closest to the actual horse body weight readings. The use of formulae for body weight estimation can be useful in determining feed dosages and additives, medicines or deworming agents. Regular weight measurement is important for maintaining a healthy horse.

## 1. Introduction

Body weight (BW) measurement and its regular recording is important in assessing the health of a horse. In young horses, regular weight measurements are very important because they provide essential information on the proper development of the animal. Weight estimation and monitoring of changes are necessary to determine the amount of feed and feed additives for the proper functioning of the animal. Measurements also provide basic information for calculating the correct dosage of medication when treatment is required, or when a deworming preparation is administered. Excessive or inadequate dosing of these agents may result in treatment failure or drug resistance [1,2,3]. A sudden change in body weight is also an important indicator of a change in health [4,5], and an increase in the weight of grazing horses in spring is closely related to the occurrence of laminitis [6,7]. Regular weight measurement and adjustment of the feeding plan based on this information are therefore very important for the maintenance of a healthy horse.

Due to the cost and practical challenge of weighing horses on a large scale, several alternative methods for estimating the body weight of horses have been developed. One of them is to determine a horse’s body weight using a formula that includes chest circumference and body length measurements. Previous research has focused on developing the most accurate equation model using this method [8,9,10,11]. In some studies, half the circumference of the chest (from the withers to the mid-abdominal line) was measured, and then the doubled value was used in the formula instead of measuring the entire circumference of the trunk. This minimizes the measurement error that may sometimes occur when the measuring tape is bent on the opposite side of the horse from the person performing the measurement [12]. In the studies conducted by Marcenac and Aublet [13], Ensminger [14], Carroll and Huntington [8], Jones et al. [10], and Martinson et al. [15], it was found that methods of estimating horse body weight using mathematical formulae have better accuracy than methods of reading BW from measuring tape, whose estimation of body weight compared to the actual weight may differ significantly [12,16]. The aim of the study was to evaluate established formulae for estimating horse body weight from data gathered using measurement tape.

Those models are developed empirically, and as such, their correspondence to the actual value of horse body weight is limited. Moreover, different breeds of horses present different body postures, which increases the possibility of errors. In consequence, the models are likely to have various reliability for different breeds. In the literature, little is known about the accuracy of the formulae, with respect to different breeds of horses. Therefore, there is a need to evaluate the error associated with employing the formulae for a different breed. The results of horse body weight, calculated by means of the formulae, were compared with the true mass of the horse measured on a weighing scale.

## 2. Materials and Methods

All horses qualified for the research were subjected to standard procedures without any harm or discomfort and therefore did not require the consent of the Local Ethical Commission for Animal Experiments at the Institute of Immunology and Experimental Therapy of the Polish Academy of Sciences in Wroclaw, Poland (Act of 15 January 2015 on protection animals used for scientific or educational purposes). Consent from all horse owners was obtained prior to the investigations.

### 2.1. Animals

The investigation was conducted on a group of 299 adult horses and ponies kept in five Horse Studs: Pepowo, Bonza, Jeziorki Osieczna, Klodzka Roza and Leka Mroczenska in Poland between July–August 2019. Five groups were specified: ponies (*n* = 58), Polish Noble Half Breed (*n* = 150), Silesian Breed (*n* = 23), Wielkopolski Breed (*n* = 52) and Thoroughbred (*n* = 16). Data were collected from horses that fulfilled the following conditions: age ≥ 3 years, BCS (Body Condition Score) of 5.0–5.5 [17,18] with 106 geldings, 155 nonpregnant mares, and 38 stallions represented. The BCS included six areas of the horse’s body (neck, withers, back, tail, ribs and back) to classify body condition on a scale of 1 (weak/poor) to 9 (extremely fat). Each area of the body was scored separately and scores were averaged to represent overall body condition. All of the horses were barefoot on all of the hooves. Included animals were housed in stables overnight and during the day were turned out on a pasture, having access to hay and water ad libitum. Currently, bred horses of the Polish Noble Half Breed and Wielkopolski Breed are maintained in Poland as a type of sports horse in the disciplines of dressage and jumping, very often as a result of mating with German sports horses of the Hanoverian, Holsteiner, or Trakehner Breeds. Horses of the Silesian Breed are characterized by a massive, harmonious conformation and long and strong, muscular neck. Horses of this breed are used mainly in carriage driving. The studied group of ponies was used in sport in the disciplines of dressage and jumping. The height at the withers of the pony was in the range of 100–146 cm.

### 2.2. Data Collection

Measurements were collected in the stable corridor on a concrete floor with an even surface. Plastic measurement tape (Zoometric Tape, Hauptner, Dietlikon-Zürich, Switzerland) with maximum measurable length 250 cm, and a measuring stick (Aluminum Horse Height Measuring Stick, Busse, Lohne, Germany) with a range 100–180 cm, were used. Trained staff performed single measurements of the horse body using the methodology described in original methodology, i.e., girth circumference at the base of the mane hairs [15], girth circumference measured over the highest point of the withers [8,14], the circumference around the abdomen at the point of the umbilicus [10], the body length from the point of buttock (tuber ischium) to the point of shoulder (head of humerus) [8,15], the length from the point of buttock to elbow (olecranon) [10,14], height at the withers (height at the third thoracic vertebra), the circumference of the neck located halfway between the poll and withers [15]. All measurements were taken by the same two persons, while a colleague held the horse. The horse limbs were always positioned parallel to each other to minimize measurement error. The true bodyweight of the horses was measured using a portable electronic scale Rhewa 82 Alpha (Rhewa Waagen, Mettmann, Germany) with a weighting platform of maximum load capacity of 1000 kg and a stated accuracy 0.5 kg.

### 2.3. Data Analysis

Statistical analysis was carried out with the use of Statistica software (v. 13.3, StatSoft Inc., Tulsa, OK, USA). The analyses were made separately for individual breeds. The investigation would only be valid if the formulae presented here gave adequate results. Therefore, in order to verify the utility of the formulae, the chi-square test and Shapiro Wilk and Kolmogorov Smirnov tests were performed on the residuals. The selection of the statistical procedure was dependent on the number of horses in the group. For data samples, fewer than 30, the Shapiro Wilk and Kolmogorov Smirnov test were performed. For greater populations, these tests do not provide accurate and reliable results. Hence, the chi-square test was performed. In both cases, the residuals were subjected to the statistical evaluation where two hypotheses were established—i.e., residuals originate from a Gaussian distribution, or alternatively, the residuals do not originate from a Gaussian distribution. The predefined formula was considered as the model, and the measured value of horse mass determined with the aid of the scale was considered as an observed value. It appeared that in the case of each considered formula there was no basis to reject the null hypothesis. Hence, the residuals could be considered to originate from the Gaussian distribution in all cases. In this sense, all the formulae could be considered for further deliberation.

The results of horse body weight measurements were compared with the formulae in Table 1. For this purpose, the differences between the observed value (i.e., true horse body weight) and the estimated value (i.e., calculated from the formulae) were determined. Additionally, the correctness of the formulae was verified by means of the root mean square error (RMSE).

## 3. Results

**Figure 1:**
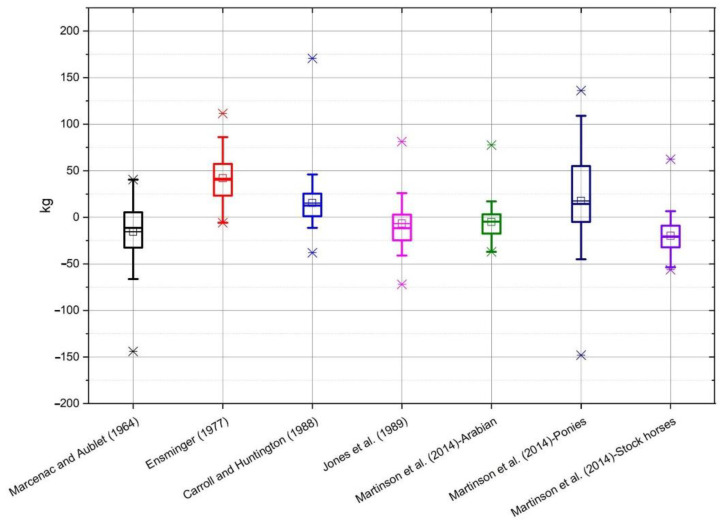
The box whiskers plot of the differences between real and estimated body weight for ponies.

**Figure 2:**
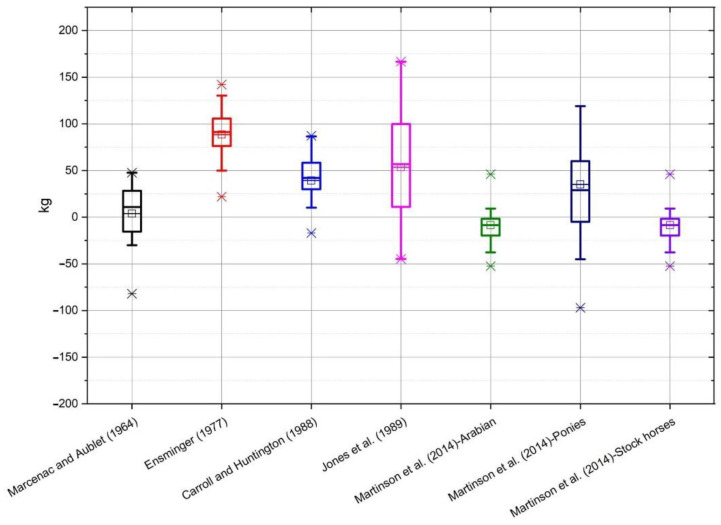
The box whiskers plot of the differences between real and estimated body weight for Silesian Breed.

**Figure 3:**
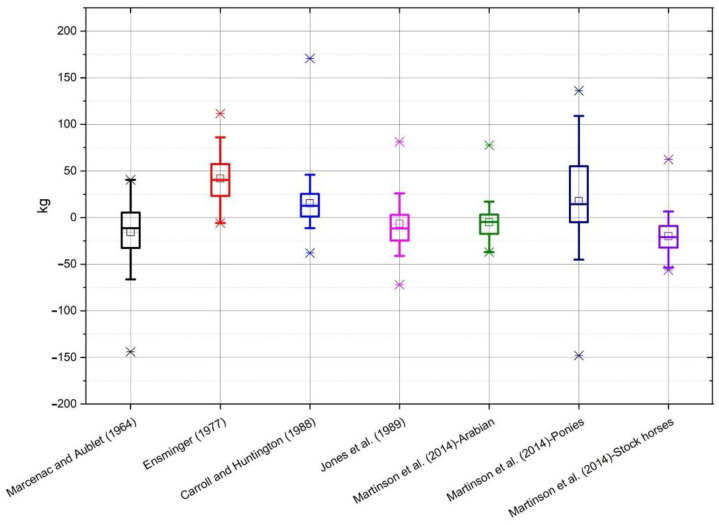
The box whiskers plot of the differences between real and estimated body weight for Polish Noble Half Breed.

**Figure 4:**
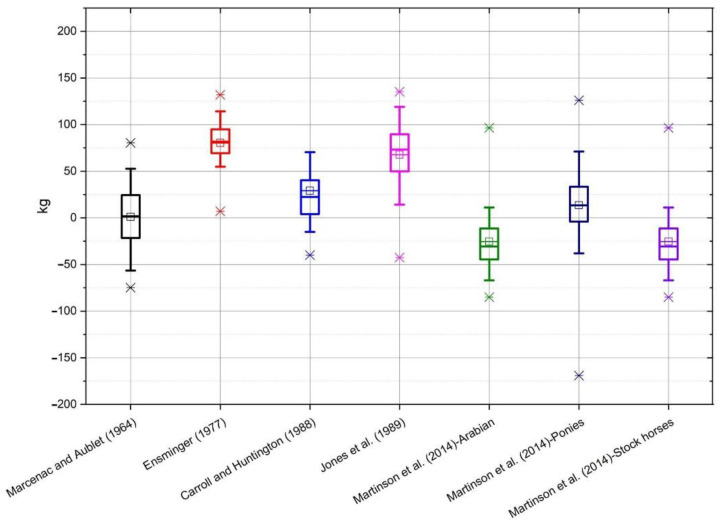
The box whiskers plot of the differences between real and estimated body weight for Wielkopolski Breed.

**Figure 5:**
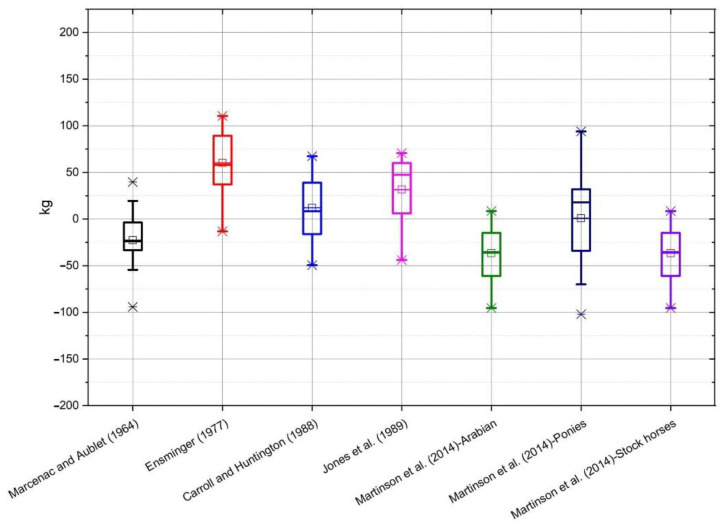
The box whiskers plot of the differences between real and estimated body weight for Thoroughbred.

On the basis of the data obtained from the conducted measurements, the bodyweight of horses was estimated according to the individual formulae (Table 1). The selected formulae were applied to all breeds of horses, and not only to those groups for which the formulae were originally developed. The average value of mass calculated using all of the formulae, as well as the measured value of mass, along with the standard deviation, is shown in Table 2. Average differences between the actual body weight and the calculated weight, depending on the breed, are shown on individual graphs (Figure 1, Figure 2, Figure 3, Figure 4 and Figure 5).

In the group of ponies (Figure 1) the most accurate formula was Martinson et al. [15] for Arabian type horses, and the least precise were Ensminger [14] and Martinson et al. [15] for ponies.

In the case of horses of Silesian Breed (Figure 2) the results of Martinson et al. [15] for Arabian type horses and stock horses were closest to the true body weight. The least accurate were Ensminger [14] and Jones et al. [10]. For Polish Noble Half Breed (Figure 3) the most accurate was Martinson et al. [15] for Arabian type horses, whereas the furthest from the true body weight were the results from Ensminger [14]. In the group of Wielkopolski Breed horses (Figure 4) the Marcenac and Aublet [13] formula proved to be the most accurate, and the least accurate were the Ensminger [14] and Jones et al. [10] formulae. The most accurate formula in the group of Thoroughbreds (Figure 5) was the Carroll and Huntington formula [8], whereas the least accurate was the formula from Ensminger [14].

**Figure 6:**
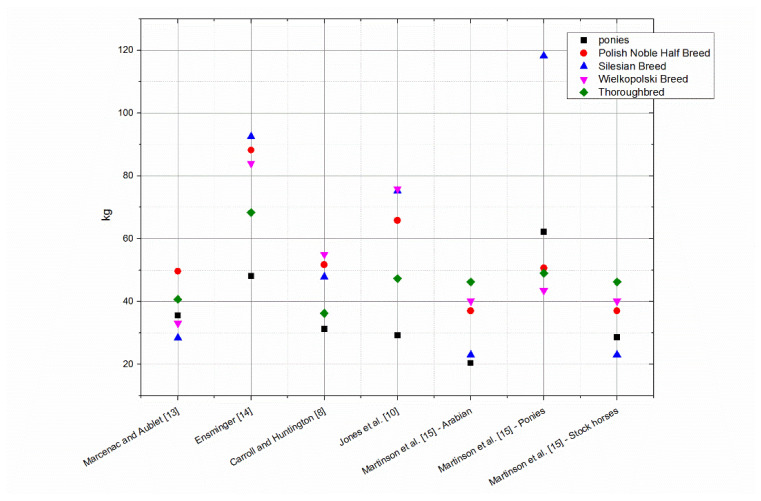
Root mean square error for individual horse groups and formulas.

The analysis of formula matching on the basis of RMSE (Figure 6) indicated that the smallest matching error exists when the bodyweight of ponies, Silesian Breed and Polish Noble Half Breed was calculated using the formula from Martinson et al. [15] designated for Arabian type horse and stock horses (Table 1). For Thoroughbreds, the smallest RMSE error was for the Caroll and Huntington [8] formula, and for horses of the Wielkopolski Breed it was the Marcenac and Aublet [13] formula.

## 4. Discussion

Research conducted on weight estimation methods by Ellis and Hollands [9] has shown that none of the methods employed to date can be recommended for the examined horse population. The research was conducted on a group of 600 horses of different breeds and ages in the UK. Bodyweight estimation from two measuring tapes, the Carroll and Huntington [8] formula, and visual bodyweight evaluation were subjected to comparison. The most accurate of the methods studied proved to be the estimation formula developed by Carroll and Huntington [8]; however, body weights significantly differed from the actual weights (*p* < 0.001). In previous studies, it was found that visual estimation of horse bodyweight was unreliable [19]. The research presented by Carroll and Huntington [8] was conducted mainly on Thoroughbreds and ponies, and suggested that the estimation formula was 90% accurate. In the experiment carried out on Hucul horses, it was shown that the most reliable procedure to estimate body weight was to use the Carroll and Huntington formula [8], which underestimated the actual body weight by an average of 7 kg, and the error of this method was 4.5% [20]. Our results show that the Carroll and Huntington [8] formula was closest to the actual body weight for Thoroughbreds.

Gharahveysi [21] conducted a study on 244 Iranian Arabian horses, demonstrating that there were no significant differences (*p* > 0.05) between the Ensminger [14] and Marcenac and Aublet [13] body weight estimation methods and a true weight, while the Jones et al. [10] formula showed a highly significant difference (*p* < 0.01). In our present study, it was found that the Marcenac and Aublet [13] formula was marked by a small relative error in Wielkopolski Breed and Silesian Breed groups. The Jones et al. [10] formula did not prove to be effective in any group.

Martinson et al. [15] examined 629 adult horses and ponies in order to develop an ideal weight estimation formula. The height at the withers and neck circumference were added to the estimation model, and all measurements could be changed by matching the additive model to the logarithmic scale of all variables. The equation obtained for body mass estimation manifested a very high correlation (r^2^ = 0.92) with true horse weight. In the present research, this formula was the most accurate method for determining bodyweight in the group of ponies, Silesian Breed, and Polish Noble Half Breed. The high accuracy of calculations based on this formula may result from taking into account not only the length of the diagonal torso and chest circumference, but also the height at the withers and the circumference of the neck. The accumulation of fat deposits increases the circumference of the thorax, and primarily the circumference of the neck [22,23]. Catalano et al. [24] also stated that adding height at the withers and neck circumference significantly improved the accuracy of the equations for estimating BW draft and warmblood horses, especially for overweight or underweight horses. In another study, they examined Miniature, saddle-type, and Thoroughbred horses, and indicated that the adding breed type, height, neck circumference, body length, and girth circumference improve BW estimation [25]. Jensen et al. [26] assessed the accuracy of various formulas for estimating bodyweight of Icelandic and warmblood horses, and also assessed the relationship between the variables for cresty neck score, BCS, and plasma concentrations of insulin. Overall, the concordance correlation coefficient was high for most formulas, but complex using at least four morphometric measurements were more accurate. Plasma insulin levels were higher (*p* < 0.001) in Icelandic horses than in warmblood horses, which was reflected in higher body fat, suggesting differences in body condition score. In our investigation, ponies and Silesian Breed horses are highly predisposed to storing fat in their neck, similar to Icelandic horses, so formulas with more measurements were more accurate.

For a more accurate estimate, the body fat and muscularity of the horse should be considered. Horse weight can vary greatly depending on the quality of work performed; horses in sport training should, in principle, be heavier due to greater muscle development. One of such equations for estimating horse weight, which includes more measurements (i.e., cannon bone circumference, neck circumference at the base) and BCS (points) is the formula developed by Kienzle and Schramme [18]. Due to ongoing breeding advances and the mixing of different horse breeds, the phenotype of horses changes. The formulae for estimating body weight may consequently need to be readjusted accordingly.

## 5. Conclusions

The Martinson et al. [15] formula recommended for Arabian type horses was the most accurate in estimating body weight in the group of ponies, Silesian Breed, and Polish Noble Half Breed, which may result from a similar fat distribution pattern in these breeds. The Marcenac and Aublet [13] formula were marked by a small relative error in the group of horses of the Wielkopolski Breed. Currently, bred horses of the Wielkopolski Breed are maintained as sports horses, very often as a result of mating with German sports horses of the Hanoverian, Holsteiner, or Trakehner Breeds. The estimations most similar to true body weight in the group of Thoroughbred horses was based on the Carroll and Huntington [8] formula.

## Figures and Tables

**Table 1:** Formulas for estimating horse body weight used in the experiment.

Reference	Application	Formula
Marcenac and Aublet [13]	adult horses	=G (m)^3^ × 80
Ensminger [14]	adult horses	=[(G (in)^2^ × L2 (in)) + 22.7]/660
Carroll and Huntington [8]	adult horses	=(G (cm)^2^ × L (cm))/11877
Jones et al. [10]	>2 year, 230 to 707 kg	=(G2 (cm)^1.78^ × L2 (cm)^0.97^)/3011
Martinson et al. [15]	Arabian type horses	=(G (cm)^1.486^ × L (cm)^0.554^ × H (cm)^0.599^ × N (cm)^0.173^)/3596
Martinson et al. [15]	ponies	=(G (cm)^1.486^ × L (cm)^0.554^ × H (cm)^0.599^ × N (cm)^0.173^)/3606
Martinson et al. [15]	stock horses	=(G (cm)^1.486^ × L (cm)^0.554^ × H (cm)^0.599^ × N (cm)^0.173^)/3441

G—girth circumference; G2—abdominal circumference on the navel; L—body length from shoulder to ischium; L2—length from elbow to ischium; H—height at withers; N—neck circumference.

**Table 2:** Average mass of horses calculated with the aid of formulae.

Formula	Application	Ponies *n* = 58	Polish Noble Half Breed *n* = 150	Silesian Breed *n* = 23	Wielkopolski Breed *n* = 52	Thoroughbred *n* = 16
		$\bar{x}\left[kg\right]$ ± SD	$\bar{x}\left[kg\right]$ ± SD	$\bar{x}\left[kg\right]$ ± SD	$\bar{x}\left[kg\right]$ ± SD	$\bar{x}\left[kg\right]$ ± SD
Marcenac and Aublet [13]	adult horses	322 ± 106	566 ± 76	584 ± 112	566 ± 65	523 ± 39
Ensminger [14]	adult horses	264 ± 86	480 ± 58	499 ± 96	487 ± 56	441 ± 37
Carroll and Huntington [8]	adult horses	291 ± 98	529 ± 65	549 ± 103	538 ± 81	489 ± 40
Jones et al. [10]	>2 year, 230 to 707 kg	313 ± 90	511 ± 67	534 ± 91	499 ± 58	469 ± 41
Martinson et al. [15]	Arabian type horses	312 ± 97	558 ± 57	571 ± 89	567 ± 64	514 ± 34
Martinson et al. [15]	ponies	311 ± 97	556 ± 37	569 ± 89	565 ± 64	513 ± 34
Martinson et al. [15]	stock horses	326 ± 02	583 ± 60	596 ± 93	592 ± 67	537 ± 35
Measured weight	N/A	306 ± 99	561 ± 63	588 ± 96	567 ± 60	501 ± 26

## References

[1] Kaushal R., Bates D.W., Landrigan C., McKenna K.J., Clapp M.D., Federico F., Goldmann D.A. (2001). Medication errors and adverse drug events in pediatric inpatients. J. Am. Med. Assoc..

[2] Powers J.H. (2009). Risk perception and inappropriate antimicrobial use: Yes, it can hurt. Clin. Infect. Dis..

[3] Runciman B., Walton M. (2007). Safety and Ethics in Healthcare: A Guide to Getting It Right.

[4] Alford P., Geller S., Richrdson B., Slater M., Honnas C., Foreman J., Robinson J., Messer M., Roberts M., Goble D. (2001). A multicenter, matched case-control study of risk factors for equine laminitis. Prev. Vet. Med..

[5] Frank N., Elliot S., Brandt L., Keisler D.H. (2006). Physical characteristics, blood hormone concentrations, and plasma lipid concentrations in obese horses with insulin resistance. J. Am. Vet. Med. Assoc..

[6] Giles S.L., Rands S.A., Nicol C.J., Harris P.A. (2014). Obesity prevalence and associated risk factors in outdoor living domestic horses and ponies. Peer J..

[7] Wylie C.E., Collins S.N., Verheyen K.L., Newton J.R. (2012). Risk factors for equine laminitis: A systematic review with quality appraisal of published evidence. Vet. J..

[8] Carroll C.L., Huntington P.J. (1988). Body condition scoring and weight estimation of horses. Equine Vet. J..

[9] Ellis J., Hollands T. (1998). Accuracy of different methods of estimating the weight of horses. Vet. Rec..

[10] Jones R.S., Lawrence T.L., Veevers A., Cleave N., Hall J. (1989). Accuracy of prediction of the liveweight of horses from body measurements. Vet. Rec..

[11] Milner J., Hewitt D. (1969). Weight of horses: Improved estimates based on girth and length. Can. Vet. J..

[12] Kyung-Nyer K. (2015). Equine Body Weight Estimation Using Three-Dimensional Images, Electronic, Scholarly Journal. Master’s Thesis.

[13] Marcenac L.N., Aublet H. (1964). Encyclopedie du Cheval.

[14] Ensminger M.E. (1977). Horses and Horsemanship.

[15] Martinson K.L., Coleman R.C., Rendahl A.K., Fang Z., McCue M.E. (2014). Estimation of body weight and development of a body weight score for adult equids using morphometric measurements. J. Anim. Sci..

[16] Hoffmann G., Bentke A., Rose-Meierhöfer S., Ammon C., Mazetti P., Hardarson G.H. (2013). Estimation of the body weight of Icelandic horses. J. Equine Vet. Sci..

[17] Henneke D.R., Potter G.D., Kreider J.L., Yeates B.F. (1983). Relationship between condition score, physical measurements and body fat percentage in mares. Equine Vet. J..

[18] Kienzle E., Schramme S.C. (2004). Beurteilung des Ernährungszustandes mittels Body Condition Scores und Gewichtsschätzung beim adulten Warmblutpferd. Pferdeheilkunde.

[19] Johnson E., Asquith R., Kivipelto J. Accuracy of weight determination of equids by visual estimation. Proceedings of the 11th ENPS.

[20] Łuszczyński J., Michalak J., Pieszka M. (2019). Assessment of methods for determining body weight based on biometric dimensions in Hucul horses. Sci. Ann. Pol. Soc. Anim. Prod..

[21] Gharahveysi S. (2012). Compare of different formulas of estimating the weight of horses by the Iranian Arab Horse data. J. Anim. Vet. Adv..

[22] Carter R.A., Geor R.J., Staniar W.B., Cubitt T.A., Harris P.A. (2009). Apparent adiposity assessed by standardised scoring systems and morphometric measurements in horses and ponies. Vet. J..

[23] Thatcher C.D., Pleasant R.S., Geor R.J., Elvinger F. (2012). Prevalence of overconditioning in mature horses in southwest Virginia during the summer. J. Vet. Intern. Med..

[24] Catalano D.N., Coleman R.J., Hathaway M.R., McCue M.E., Rendahl A.K., Martinson K.L. (2016). Estimation of actual and ideal bodyweight using morphometric measurements and owner guessed bodyweight of adult draft and warmblood horses. J. Equine Vet. Sci..

[25] Catalano D.N., Coleman R.J., Hathaway M.R., Neu A.E., Wagner E.L., Tyler P.J., McCue M.E., Martinson K.L. (2019). Estimation of actual and ideal bodyweight using morphometric measurements of Miniature, saddle-type, and Thoroughbred horses. J. Equine Vet. Sci..

[26] Jensen R.B., Rockhold L.L., Tauson A.H. (2019). Weight estimation and hormone concentrations related to body condition in Icelandic and Warmblood horses: A field study. Acta Vet Scand..

